# Effect of COVID-19 Pandemic on Admission Pattern to Pediatric Surgery Division at King Fahad University Hospital and Subsequent Quality of Presentation in Cases: A Comparative Study and Single-Center Experience

**DOI:** 10.7759/cureus.66305

**Published:** 2024-08-06

**Authors:** Njoud J Alsunnary, Lujain Al Turkistani, Shareefa Alhemaid, Fatimah Al Shehab, Maryam Al Hashimi, Hossam Elshafei, Hussah M Al-Buainain, Rawan A Alkhudaimi, Husain N Alshaikh

**Affiliations:** 1 Medical School, Imam Abdulrahman Bin Faisal University, Dammam, SAU; 2 Surgery, King Fahad University Hospital, Dammam, SAU; 3 Pediatric Surgery, King Fahad University Hospital, Dammam, SAU

**Keywords:** surgical severity, inguinal hernia, appendicitis, pediatric surgery, covid-19

## Abstract

The coronavirus disease 2019 (COVID-19) pandemic has affected healthcare systems worldwide, with mandatory quarantine and isolation measures being implemented to curb the spread of the virus. These measures have potentially led to delayed or complicated presentations of non-COVID-19 cases, including pediatric surgical cases. This study aims to evaluate pediatric surgical admission patterns, analyze the incidence of surgical diagnoses, and assess the severity of presentation during the COVID-19 period compared to the pre-COVID-19 period. This retrospective observational study was conducted at a university hospital in the eastern region of Saudi Arabia to assess the effect of the pandemic on pediatric surgery admissions patterns and severity of presentation during the COVID-19 period (March 2, 2020, to March 1, 2022) and pre-COVID-19 period (March 1, 2018, to March 1, 2020). Of the 903 pediatric surgical admissions, 366 (40.5%) presented during the COVID-19 period. The admission rate per month decreased by 6.9 during COVID-19 compared to pre-COVID-19 (mean [SD]: 21.5 [9.3] vs. 14.6 [8.2], p = 0.01). The most common admission diagnoses were appendicitis (17.5%) and inguinal hernia (15.8%). There was a 15% increase in the percentage of emergency admissions (54.4% vs. 47.3%, p = 0.037) during COVID-19 compared to pre-COVID-19. Of note, the percentage of patients admitted with acute appendicitis increased by 35.9% (20.8% vs. 15.3%, p = 0.03). Furthermore, the emergency admissions for patients with inguinal hernia doubled (26.6% vs. 12.7%, p = 0.035). No significant difference in ICU admissions, hospital length of stay, and routine discharge were observed. In conclusion, the COVID-19 pandemic correlated with a significant decrease in overall admissions and an increase in emergency admissions, including those for appendicitis and inguinal hernia. The increase in complicated conditions was not significant. There was no significant difference in ICU admissions and hospital length of stay. Future studies involving multiple centers are necessary to validate these findings.

## Introduction

The severe acute respiratory syndrome coronavirus 2 (SARS-CoV2) virus causing atypical pneumonia termed coronavirus disease 2019 (COVID-19) began as an epidemic in China in December 2019 and rapidly spread to neighboring countries. By the beginning of March 2020, it had spread globally and had reached Saudi Arabia [1]. In order to curb the further spread of the virus, the Saudi government implemented mandatory quarantine and isolation measures for infected individuals. Additionally, a curfew was imposed, limiting access to healthcare facilities. Concerned guardians have also been hesitant to seek medical attention for their children, fearing the risk of COVID-19 infection [2]. As a result, non-COVID-19 health issues, whether mild or severe, have been delayed, potentially leading to avoidable complications and significantly impacting the quality of pediatric presentations and outcomes [3-5].

In addition to these governmental measures, local hospitals have taken special measures to secure the resources required to provide care for COVID-19 patients throughout the pandemic. These measures included the establishment of additional intensive care units (ICU), dedicated wards, and clinics for COVID-19 patients. Furthermore, hospital administration suspended elective medical care, including surgical procedures, throughout the COVID-19 period. These measures may have contributed to delayed or complicated presentations of non-COVID-19 cases.

As previously mentioned, COVID-19 had a major effect on all aspects of the healthcare system. Various studies in both adults and pediatrics have reported a remarkable reduction in hospital admissions, emergency consultations, and ICU admissions during the pandemic [2,6-12]. However, only a few addressed the effects on pediatric patients with surgical presentations. In particular, there is a lack of research on surgical cases during COVID-19 in Saudi Arabia, with very few studies available on this topic.

The main aim of this study was to evaluate the impact of COVID-19 on admission patterns to the pediatric surgery department, analyze the incidence of surgical diagnoses, and assess the severity of presentation during the COVID-19 period compared to the pre-COVID-19 period.

## Materials and methods

Data source, study design, and selection criteria

This single-center retrospective observational study was conducted at a university hospital in the eastern region of Saudi Arabia. We queried the hospital’s digital medical record database (QuadraMed, Harris Healthcare, Plano, TX) from March 1, 2018, to March 1, 2022, for all hospital encounters under the care of the pediatric surgery service. We included all elective and emergency encounters that required hospital admissions. The pediatric patient population was set for all individuals aged 14 years or younger per hospital policy. Hospital encounters were divided by the date of presentation into two comparison groups: pre-lockdown (March 1, 2018, to March 1, 2020) and during lockdown (March 2, 2020, to March 1, 2022). This dichotomy point was made to coincide with the date when elective admissions and utilization of the operative room were drastically restricted due to the COVID-19 pandemic. All encounters met the inclusion criteria, except for seven encounters that were excluded from analysis due to incomplete medical record data.

Variable definitions

The collected data comprised demographic and baseline characteristics, as well as surrogate variables for the presentation severity. These included age, sex, nationality, past medical and surgical history, admission diagnosis, primary and secondary procedures, imaging, and SARS-CoV-2 test results. Furthermore, we included variables for the most commonly performed procedures in our institution. This included laparoscopic appendectomy, abscess incision and drainage, open inguinal hernia repair, orchidopexy, and circumcision. The latter was further divided into two types: standalone (performed as an independent procedure) and combined (performed in combination with other index procedures like orchidopexy or inguinal hernia repair). The complicated presentation for the most common surgical diagnoses was defined as follows. Complicated appendicitis included patients who were found to have appendiceal perforation, suppuration, gangrene, and/or phlegmon. As for inguinal hernia, it was considered complicated if it was associated with irreducibility (incarceration), obstruction, or strangulation. The duration, in days, for admissions to the ICU was also recorded. Routine discharge was defined as any patient who was not discharged against medical advice (DAMA), rescheduled, or deceased. Antibiotics were classified into two distinct categories: (i) narrow spectrum, which included cefazolin, ceftriaxone, gentamicin, clindamycin, amoxicillin/clavulanate, and metronidazole, and (ii) wide spectrum, which included piperacillin/tazobactam, meropenem, and vancomycin.

Statistical analysis

Continuous variables were summarized using median with interquartile range (IQR) or means and standard deviations, while categorical variables were summarized using count and percentages. The Mann-Whitney Wilcoxon test and student’s t-test were used to compare continuous variables as appropriate, while the chi-square test was used to compare categorical variables. In addition, linear regression was used to identify the average number of admissions per month before and during the COVID-19 lockdown/restriction period. Statistical significance was set at a p-value of <0.05. The study used Stata 13.1 (StataCorp., College Station, TX) to perform data management and analyses.

Ethical consideration

Ethical approval was obtained from the King Fahad Hospital of the University (Al Khobar, Saudi Arabia) Institutional Review Board (IRB-UGS-2022-01-424). Informed consent from patients was not required during the data collection process, and measures to ensure patient data confidentiality were instated.

## Results

Table 1 shows the baseline characteristics and clinical data of pediatric patients included in both pre-COVID-19 (2018-2020) and during COVID-19 (2020-2022) study periods. A total of 366 (40.5%) pediatric patients were admitted during the COVID-19 study period from March 2, 2020, to March 1, 2022, compared with 537 (59.5%) patients in the pre-COVID-19 period from March 1, 2018, to March 1, 2020. This indicates a reduction in hospitalization by 19% during the COVID-19 crisis. The majority of the admitted pediatric patients were males (69%) with a median age of 4 years in both study periods. During the COVID-19 period, 2.5% of patients tested positive for COVID-19. The number of sickle cell disease (SCD) patients’ hospital admissions significantly increased from 1.3% to 3.8% (p = 0.014) during the COVID-19 period, and an increase in thalassemia patients’ presentation was noted by a significant 1.8% (p = 0.011). However, there was no remarkable change in glucose-6-phosphate dehydrogenase deficiency (G6PD) and asthma patients’ admissions between pre-COVID-19 and during COVID-19 study periods. Regarding admission diagnoses, the most prevalent diagnosis in both study periods was appendicitis, with a total number of 158 (17.1%) cases, followed by inguinal hernia cases of 143 (15.8%). Appendicitis cases had a significant rise from 15.3% to 20.8% (p = 0.033), while the increase in inguinal hernia cases from 14.7% to 17.5% wasn’t statistically significant (p = 0.262). Moreover, cases of abscess, foreign body ingestion, trauma, and other types of hernia didn’t evidently change between pre-COVID-19 and during COVID-19 periods. Appendectomy was the only procedure that increased significantly during the COVID-19 period, from 14.2% to 19.1 (p = 0.046). On the other hand, the number of herniotomy procedures had increased from 12.8% to 16.4%, though it was not statistically significant (p = 0.135), while other procedures, including circumcision, incision and drainage, and orchiopexy, had no significant differences between pre-COVID-19 and during COVID-19 periods. 

**Table 1:** Baseline characteristics, count, and percentages in the pre-COVID-19 period (2018-2020) and during the COVID-19 period (2020-2022) PSH, past surgical history; PMH, past medical history

	Pre-COVID, n (%)	During COVID, n (%)	Total, n (%)	p-value
Count (row %)	537 (59.5)	366 (40.5)	903 (100)	
Age (days), median (IQR)	1460 (510-3285)	1460 (360-3285)	1460 (450-3285)	0.737
Female	167 (31.1)	115 (31.4)	282 (31.2)	0.918
Non-Saudi	57 (10.6)	37 (10.1)	94 (10.4)	0.807
Positive PSH	68 (12.7)	44 (12.0)	112 (12.4)	0.774
Positive PMH	126 (23.5)	96 (26.2)	222 (24.6)	0.343
PMH				
Asthma	44 (8.2)	28 (7.7)	72 (8.0)	0.767
SCD	7 (1.3)	14 (3.8)	21 (2.3)	0.014
G6PD	6 (1.1)	4 (1.1)	10 (1.1)	0.973
Thalassemia	2 (0.4)	8 (2.2)	10 (1.1)	0.011
COVID test				
Not done	526 (98.0)	10 (2.7)	536 (59.4)	<0.001
Negative	11 (2.0)	347 (94.8)	358 (39.6)	
Positive	0 (0.0)	9 (2.5)	9 (1.0)	
Admission diagnosis				
Appendicitis	82 (15.3)	76 (20.8)	158 (17.5)	0.033
Inguinal hernia	79 (14.7)	64 (17.5)	143 (15.8)	0.262
Abscess (any)	34 (6.3)	21 (5.7)	55 (6.1)	0.714
Trauma	23 (4.3)	23 (6.3)	46 (5.1)	0.179
Other hernia	16 (3.0)	12 (3.3)	28 (3.1)	0.799
Foreign body ingestion	9 (1.7)	7 (1.9)	16 (1.8)	0.791
Procedures (total)	407 (75.8)	296 (80.9)	703 (77.9)	0.071
Appendectomy	76 (14.2)	70 (19.1)	146 (16.2)	0.046
Herniotomy	69 (12.8)	60 (16.4)	129 (14.3)	0.135
Circumcision (all)	66 (12.3)	47 (12.8)	113 (12.5)	0.806
Circumcision (standalone)	31 (5.8)	21 (5.7)	52 (5.8)	0.982
Incision & drainage	30 (5.6)	18 (4.9)	48 (5.3)	0.66
Orchidopexy	24 (4.5)	17 (4.6)	41 (4.5)	0.901
Imaging (overall)	312 (58.1)	222 (60.7)	534 (59.1)	0.443
X-ray	180 (33.5)	127 (34.7)	307 (34.0)	0.713
Ultrasound	185 (34.5)	132 (36.1)	317 (35.1)	0.618
CT scan	81 (15.1)	67 (18.3)	148 (16.4)	0.199
MRI	24 (4.5)	12 (3.3)	36 (4.0)	0.369

Table 2 shows the difference in the severity of presentation of surgical pediatric admissions between the pre-COVID-19 period (2018-2020) and during the COVID-19 period (2020-2022). There was a significant increase in the number of emergency admissions. It increased from 47.3% in the pre-COVID-19 period to 54.4% during the COVID-19 period (p = 0.037). In addition, there was a significant increase in antibiotic use for both narrow and broad-spectrum antibiotics. There was a 4.2% increase in the use of narrow-spectrum antibiotics and a 3% increase in the use of broad-spectrum antibiotics (p = 0.047). However, there was no difference in antibiotic use duration before and during the COVID-19 period. Moreover, there was a 3.1% increase in the number of complicated diagnoses, but it was not statistically significant (p = 0.133). Furthermore, the number of ICU admissions, length of hospital stay, and routine discharge showed no significant difference in both periods.

**Table 2:** Severity of presentation (overall), count, and percentages in the pre-COVID-19 period (2018-2020) and during the COVID-19 period (2020-2022)

	Pre-COVID, n (%)	During COVID, n (%)	Total, n (%)	p-value
Emergency	254 (47.3)	199 (54.4)	453 (50.2)	0.037
Antibiotic use				
No	291 (54.2)	172 (47.0)	463 (51.3)	0.047
Yes (narrow spectrum)	218 (40.6)	164 (44.8)	382 (42.3)	
Yes (broad spectrum)	28 (5.2)	30 (8.2)	58 (6.4)	
Abx use duration (days), median (IQR)	7 (3-9)	7 (2-10)	7 (3-10)	0.219
ICU admission	21 (3.9)	19 (5.2)	40 (4.4)	0.358
Complicated disease	48 (8.9)	44 (12.0)	92 (10.2)	0.133
Routine discharge	516 (96.1)	352 (96.2)	868 (96.1)	0.948
Length of stay, median (IQR)	2 (1-4)	2 (1-3)	2 (1-4)	0.111

Table 3 shows the difference in the severity of presentation of acute appendicitis between the pre-COVID-19 period (2018-2020) and during the COVID-19 period (2020-2022). The number of emergency admissions for acute appendicitis increased by 2.4%, although this difference was not statistically significant (p = 0.171). Additionally, the number of complicated appendicitis increased by 10.5%. However, the difference was also not significant (p = 0.110). Moreover, there was a non-significant decrease of 2.4% in the number of ICU admissions during the COVID-19 period (p = 0.171). Furthermore, there was no significant difference between the two periods in terms of number of appendectomy procedures, age of presentation, use of antibiotics, duration of antibiotics use, length of hospital stay, and routine discharge for acute appendicitis.

**Table 3:** Severity of presentation (appendicitis), count, and percentages in the pre-COVID-19 period (2018-2020) and during the COVID-19 period (2020-2022)

	Pre-COVID, n (%)	During COVID, n (%)	Total, n (%)	p-value
Appendicitis, count (row %)	82 (51.9)	76 (48.1)	158 (100)	
Age (years), median (IQR)	10 (8-12)	10 (8-11)	10 (8-11)	0.338
Emergency	80 (97.6)	76 (100.0)	156 (98.7)	0.171
Appendectomy	75 (91.5)	68 (89.5)	143 (90.5)	0.67
Antibiotic use				
No	3 (3.7)	2 (2.6)	5 (3.2)	0.63
Yes (narrow spectrum)	71 (86.6)	63 (82.9)	134 (84.8)	
Yes (broad spectrum)	8 (9.8)	11 (14.5)	19 (12.0)	
Abx use duration (days), median (IQR)	5 (3-9)	5 (2-10)	5 (2-10)	0.822
ICU admission	2 (2.4)	0 (0.0)	2 (1.3)	0.171
Complicated appendicitis	14 (17.1)	21 (27.6)	35 (22.2)	0.11
Length of stay, median (IQR)	4 (3-6)	3 (2-5)	4 (3-5)	0.638
Routine discharge	81 (98.8)	75 (98.7)	156 (98.7)	0.957

Table 4 shows the difference in the quality/severity of presentation of inguinal hernia between the pre-COVID-19 period (2018-2020) and during the COVID-19 period (2020-2022). There was a significant increase in the number of emergency admissions for inguinal hernia during the COVID-19 period, with emergency admissions rising from 12.7% to 26.6% (p = 0.035). Additionally, the difference in the number of complicated inguinal hernias between the two periods was marginally significant, and it increased from 11.4% to 23.4% during the COVID-19 period (p = 0.055). Furthermore, there was no significant difference in the number of herniotomy procedures, use of antibiotics, duration of antibiotic use, number of ICU admissions, length of hospital stay, and routine discharge for inguinal hernia between the two periods.

**Table 4:** Severity of presentation (inguinal hernia), count, and percentages in the pre-COVID-19 period (2018-2020) and during the COVID-19 period (2020-2022)

	Pre-COVID, n (%)	During COVID, n (%)	Total, n (%)	p-value
Inguinal hernia, count (row %)	79 (55.2)	64 (44.8)	143 (100)	
Age (years), median (IQR)	1 (0-4)	1 (0-2)	1 (0-3)	0.204
Emergency	10 (12.7)	17 (26.6)	27 (18.9)	0.035
Herniotomy	67 (84.8)	58 (90.6)	125 (87.4)	0.297
Antibiotic use				
No	69 (87.3)	50 (78.1)	119 (83.2)	0.221
Yes (narrow spectrum)	8 (10.1)	13 (20.3)	21 (14.7)	0.221
Yes (broad spectrum)	2 (2.5)	1 (1.6)	3 (2.1)	0.221
Antibiotic use duration (days), median (IQR)	6 (4-10)	2 (1-8)	4 (1-8)	0.239
ICU admission	2 (2.5)	3 (4.7)	5 (3.5)	0.485
Complicated inguinal hernia	9 (11.4)	15 (23.4)	24 (16.8)	0.055
Length of stay, median (IQR)	1 (1-3)	1 (1-2)	1 (1-3)	0.553
Routine discharge	69 (87.3)	61 (95.3)	130 (90.9)	0.099

**Figure 1:**
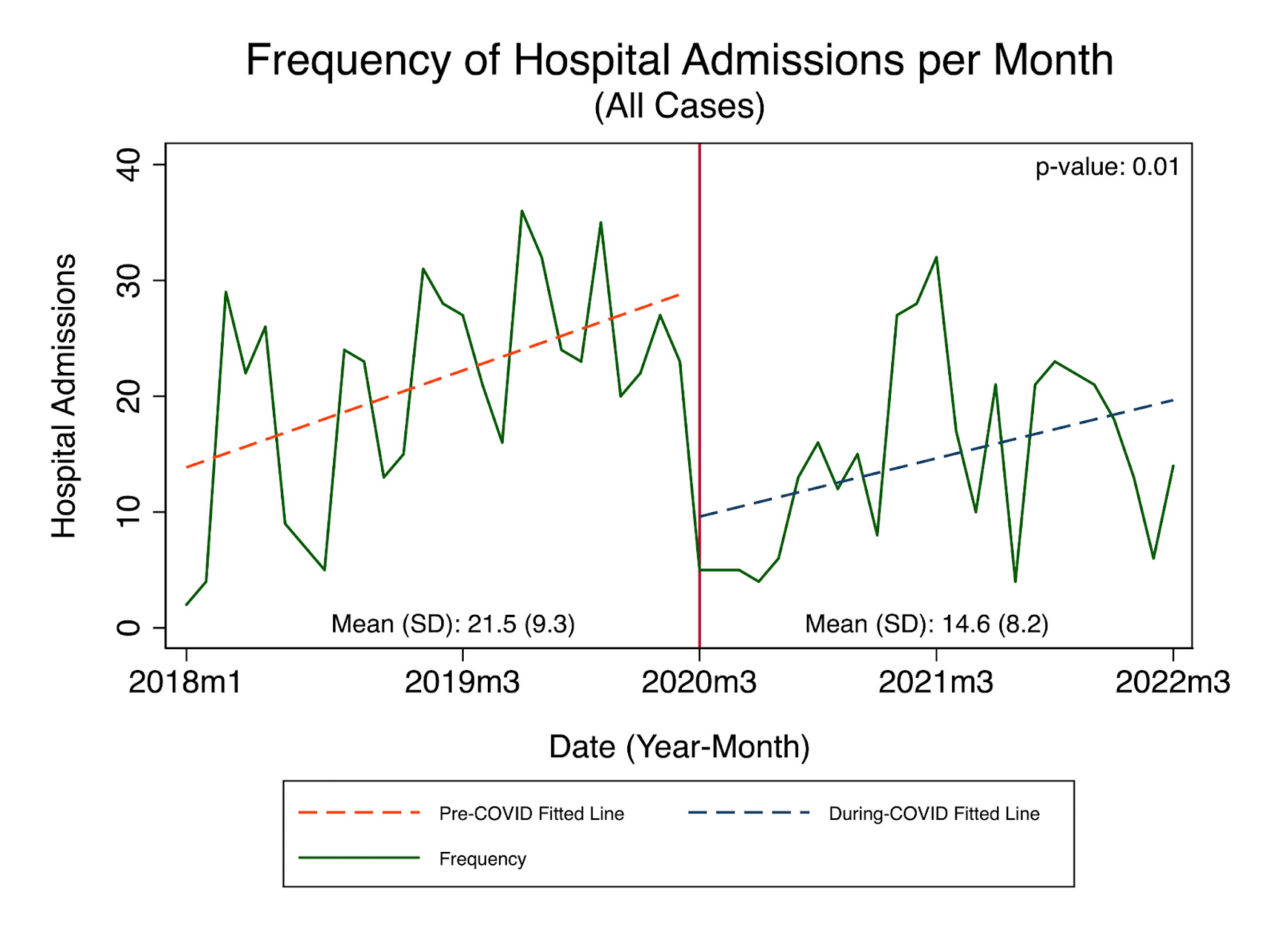
Frequency of hospital admissions in the pre-COVID-19 period (March 2018-March 2020) compared to during the COVID-19 period (March 2020-March 2022)

Figure 1 shows the frequency of hospital admissions in the pre-COVID-19 period (March 2018-March 2020) compared to during the COVID-19 period (March 2020-March 2022). There was a significant decline in the frequency of hospital admissions during the COVID-19 period compared to the pre-COVID-19 period. Approximately the average rate of hospital admissions recorded in pre-COVID was 21.5 admissions per month compared to 14.6 admissions per month (p = 0.01). However, the frequency of hospital admissions peaked during the COVID-19 period after the partial reopening of outpatient clinics in October 2020, and then it was followed by a significant reduction of admissions after the re-implication of restrictions and reduction of all elective cases in April 2021.

## Discussion

In this study, we analyzed pediatric emergency surgical admissions during and before the COVID-19 pandemic and compared the severity of the presentation between the two periods. The overall number of hospital admissions significantly decreased during the pandemic, with appendicitis being the most prevalent diagnosis, followed by inguinal hernia. In addition, appendicitis cases have notably increased during the pandemic. Furthermore, the pandemic moderately impacted the severity of cases as the emergency admissions count increased with a marginal rise in complicated cases. Regarding inguinal hernias, emergency admissions have also risen significantly in the COVID-19 era, with a slight rise in complicated inguinal hernias.

Pediatric surgical admissions for non-COVID-19-related diagnoses have had a notable decrease during the COVID-19 pandemic compared to previous years. This decline may be attributed to several factors, including the guardians’ fear of their children contracting COVID-19 within hospitals, the implementation of lockdown measures, and the closure of elective admissions, along with the re-allocation of resources, which includes the designation of pediatric surgery wards for COVID-19 admissions.

Despite the decline in overall pediatric surgical admissions, emergency admissions have increased significantly during the COVID-19 pandemic. One possible explanation for this trend is that our center continued to provide care to non-COVID-19 emergency cases, whereas other centers dedicated their resources to COVID-19 patients only. This potentially led to a higher number of patients seeking emergency care at our hospital. Additionally, the delay in seeking medical attention may have led to more severe cases requiring emergency care. Specifically, the admission rate for acute urgent diagnoses such as appendicitis has significantly increased, likely due to the urgent nature of the condition and the need for immediate surgery based on the severity of its presentation. We have expected an increase in the overall severity of presentation during the COVID-19 period. However, our data suggest that the severity of presentation during COVID-19 admissions, represented by ICU admissions, length of hospital stay, and routine discharge, is comparable to previous years, which is consistent with existing literature [4]. This may suggest that despite the lockdown measures, there were no significant access challenges to our hospital and that our hospital protocols were able to maintain the quality of care for admitted patients despite the challenging circumstances. Interestingly, the use of antibiotics has increased significantly during the COVID-19 period. This increase may be attributed to the higher number of patients presenting with comorbidities or other associated pathologies requiring antibiotics use.

Furthermore, all cases of acute appendicitis patients underwent appendectomy during the COVID-19 period, except 10.5% that either were not fit for the surgery due to mass or abscess and/or had been treated conservatively. An open appendectomy approach was recommended during the pandemic to minimize aerosol generation. However, after our hospital’s internal audit, the pediatric surgical department opted to continue with a laparoscopic approach as there was no solid clinical evidence in support of changing the practice. Nonetheless, proper precautions were maintained, such as wearing personal protective equipment (PPE). Although insignificant, a higher rate of complicated appendicitis has been found during the COVID-19 period. This rate may be explained by the implementation of the curfew, as well as parents’ fears of getting infected by the virus, leading to delayed hospital visits. An insignificant increase was also found in a study done in the United States [13]. However, other studies found a significant increase in complicated appendicitis [13-15]. Interestingly, our results show no significant difference in hospitalization length and antibiotic use duration between the two periods in contrast to the results of other studies [13-15].

At our center, patients with inguinal hernia presenting with an attack of irreducibility were previously referred to the pediatric surgery clinic for scheduling an elective procedure if reduced. However, during the pandemic and limitations due to mandated curfew, any patient presenting with an inguinal hernia to the emergency department was admitted for herniotomy. The change in standard practice, along with the closure of elective procedures, would explain the increase in emergency admission of inguinal hernia cases from 12.7% to 26.6% during the pandemic. It was expected that all admitted cases would have undergone surgical intervention. However, it was found that six patients (9.4%) had not undergone surgery. This was mostly due to the preference of guardians and surgeons of patients to either undergo a herniotomy or the patient be discharged and rescheduled electively once restrictions eased. The choice is made available for non-urgent cases that are reducible and uncomplicated, especially if these patients are COVID-19 positive. Moreover, there was an increase in complicated inguinal hernia cases from 11.4% to 23.4% during the COVID-19 period. This increase is likely attributed to the previously mentioned factors that may result in a delay in presentation.

Limitations and recommendations

The main limitation of this study, which needs to be addressed in future research, is that it was conducted in a hospital that was assigned to be a COVID-19 center during the pandemic. This makes the number of hospital admissions of non-COVID-19 patients decline significantly during the pandemic, which has affected the study sample and, subsequently, the statistical results. Furthermore, the study was conducted in a single tertiary center, rendering the generalizability restricted to equivalent institutions. In addition, due to the retrospective design of our study, the data collected from previously recorded information in the medical chart review may have contained missing information. As a result, it has led to the exclusion of patients, further reducing the sample size. Future studies on a larger scale involving multiple centers across the country are necessary to provide further validation of these findings.

## Conclusions

In conclusion, the COVID-19 pandemic has had a major impact on pediatric surgical admissions and quality of presentation. During the COVID-19 period, the number of overall admissions decreased while the number of emergency admissions, including those for appendicitis and inguinal hernia, increased. This can be attributed to various factors, including the closure of elective surgeries, avoidance of care for fear of contracting the virus, reallocation of resources toward COVID-19 care, and mandatory lockdown and curfew. Although the number of complicated conditions has increased, this increase is not significant and is comparable to the pre-COVID-19 period. This may suggest that despite the pandemic’s challenges, access to care at the hospital was not significantly impacted. In addition, no significant difference in ICU admissions and hospital length of stay was observed. This may indicate that the hospital’s efforts and protocols were able to maintain the quality of care despite hospital disruptions.

This study can provide valuable insight into the effect of the pandemic on pediatric health and surgical admissions. It can help guide future healthcare policies and allocation of resources regarding public health crises and pandemic outbreaks. Future studies on a larger scale involving multiple centers across the country are necessary to provide further validation of these findings.
